# A comparison of admission and worst 24-hour Acute Physiology and Chronic Health Evaluation II scores in predicting hospital mortality: a retrospective cohort study

**DOI:** 10.1186/cc3913

**Published:** 2005-11-25

**Authors:** Kwok M Ho, Geoffrey J Dobb, Matthew Knuiman, Judith Finn, Kok Y Lee, Steven AR Webb

**Affiliations:** 1 Consultant Intensivist Department of Intensive Care, Royal Perth Hospital, Wellington street, Perth, WA 6000, Australia; 2 PhD candidate, School of Population Health, University of Western Australia, Crawley, Perth, WA 6009, Australia; 3 PhD candidate, School of Medicine and Pharmacology, University of Western Australia, Crawley, Perth, WA 6009, Australia; 4 Acting Head of the Department, Department of Intensive Care, Royal Perth Hospital, Wellington street, Perth, WA 6000, Australia; 5 Associate Professor, School of Medicine and Pharmacology, University of Western Australia, Crawley, Perth, WA 6009, Australia; 6 Professor, School of Population Health, University of Western Australia, Crawley, Perth, WA 6009, Australia; 7 Senior Lecturer, School of Population Health, University of Western Australia, Crawley, Perth, WA 6009, Australia; 8 Consultant Intensivist, Department of Intensive Care, Royal Perth Hospital, Wellington street, Perth, WA 6000, Australia; 9 Senior Lecturer, School of Medicine and Pharmacology, University of Western Australia, Crawley, Perth, WA 6009, Australia

## Abstract

**Introduction:**

The Acute Physiology and Chronic Health Evaluation (APACHE) II score is widely used in the intensive care unit (ICU) as a scoring system for research and clinical audit purposes. Physiological data for calculation of the APACHE II score are derived from the worst values in the first 24 hours after admission to the ICU. The collection of physiological data on admission only is probably logistically easier, and this approach is used by some ICUs. This study compares the performance of APACHE II scores calculated using admission data with those obtained from the worst values in the first 24 hours.

**Materials and Methods:**

This was a retrospective cohort study using prospectively collected data from a tertiary ICU. There were no missing physiological data and follow-up for mortality was available for all patients in the database. The admission and the worst 24-hour physiological variables were used to generate the admission APACHE II score and the worst 24-hour APACHE II score, and the corresponding predicted mortality, respectively.

**Results:**

There were 11,107 noncardiac surgery ICU admissions during 11 years from 1 January 1993 to 31 December 2003. The mean admission and the worst 24-hour APACHE II score were 12.7 and 15.4, and the derived predicted mortality estimates were 15.5% and 19.3%, respectively. The actual hospital mortality was 16.3%. The overall discrimination ability, as measured by the area under the receiver operating characteristic curve, of the admission APACHE II model (83.8%, 95% confidence interval = 82.9–84.7) and the worst 24-hour APACHE II model (84.6%, 95% confidence interval = 83.7–85.5) was not significantly different (*P *= 1.00).

**Conclusion:**

Substitution of the worst 24-hour physiological variables with the admission physiological variables to calculate the admission APACHE II score maintains the overall discrimination ability of the traditional APACHE II model. The admission APACHE II model represents a potential alternative model to the worst 24-hour APACHE II model in critically ill nontrauma patients.

## Introduction

Scoring systems such as Acute Physiology and Chronic Health Evaluation (APACHE), the Therapeutic Intervention Scoring System, and Mortality Probability Models (MPM) have been developed and used as quality assurance tools and for risk stratification in research involving critically ill patients [1,2]. Each scoring system has its own strengths and weaknesses, and the choice depends on the system's ease of use and goodness of fit for that particular intensive care unit (ICU) or patient group.

The traditional APACHE II model utilises the worst values of 12 physiological variables during the first 24 hours following ICU admission, along with an evaluation of the patient's chronic health and admission diagnosis to calculate the APACHE II predicted mortality [3]. The APACHE II model has been widely validated and used by many ICUs to classify the severity of illness and to predict hospital mortality [2,4-7]. APACHE II has now been modified to APACHE III; however, some studies have shown that APACHE III may underestimate the number of deaths [8,9]. Although the APACHE II model is quite old, and other scoring systems have been developed using more recent cohorts, APACHE II is still widely used for research and clinical audit purposes. APACHE II is easier to use than APACHE III and has been in use for a long period, which allows consistency [2,10].

A potential problem with these methods is that the worst 24-hour physiological data used to derive APACHE II scores and APACHE III scores can be treatment-dependent and therefore it may reflect poor clinical management rather than sicker patients [11-13]. Collection of the admission physiological variables rather than the worst 24-hour physiological variables is a standard practice in some ICUs to calculate the APACHE II predicted mortality, and may theoretically overcome this potential problem [14,15]. The use of only admission physiological variables may make data collection easier as the data collector does not need to peruse all the blood tests and physiological variables over 24 hours to work out the worst score. However, the performance of APACHE II scores using admission data has not been thoroughly assessed [3,16].

When the APACHE III scoring system was developed, the effect of using admission physiological variables rather than the worst 24-hour physiological variables was assessed. The absolute difference between the mean scores, derived from the admission and worst 24-hour physiological data, was not statistically significantly different from zero [16]. However, the proportion of missing values favoured the worst 24-hour values over the admission values, as did the maximum explanatory power. Some other scoring systems use only admission data (MPM II_0 _and Simplified Acute Physiology Score [SAPS] III), and it is therefore established that scoring systems using physiological data from the time of admission to the ICU can provide valid assessment of the severity of illness and outcome prediction [17,18].

In the present study we evaluated the performance of the APACHE II model using physiological data at the time of ICU admission with the model using data obtained from the worst values in the first 24 hours.

## Materials and methods

This was a retrospective cohort study that utilised prospectively collected data. The study was conducted in the medical–surgical ICU at Royal Perth Hospital, an 800-bed university teaching hospital. The 22-bed ICU is a 'closed' ICU that admits critically ill adult patients of all specialties and is staffed by fully trained intensivists. The unit database contains de-identified information for components of the APACHE II score for physiological data collected at admission and for the worst values in the first 24 hours – admission diagnosis and source, age, ethnicity, ICU mortality and hospital mortality. The admission and the worst 24-hour physiological data were used to generate the admission APACHE II score and the worst 24-hour APACHE II score, respectively. The admission APACHE II score and the worst 24-hour APACHE II score were then used to calculate the admission APACHE II predicted mortality (admission APACHE II model) and the worst 24-hour predicted mortality (worst 24-hour APACHE II model), using the published APACHE II mortality prediction equation coefficients [3].

The data were collected by the duty ICU consultant on paper sheets and updated on a daily basis by the duty consultant while the patient remained in the ICU. After the patient was discharged from the ICU, the data were checked for transcription errors and completeness by a designated trained clerical staff member using data from the computerised laboratory database, going through the ICU vital signs flow chart again before the data were transferred to the computer. A total of 12 consultants were involved in collecting data, of which seven were involved throughout the study period, using a standardised data dictionary. The worst 24-hour APACHE II score was determined precisely as described by Knaus and colleagues [3].

Measurement of all 12 physiological variables on admission and over the first 24 hours in the ICU was mandatory in the APACHE data recording form. If the patient was anaesthetised before ICU admission, the Glasgow coma score was assessed using the available clinical information prior to anaesthesia. Acute renal failure was defined as oliguria with urine output less than 135 ml over a consecutive 8-hour period with abnormal serum creatinine concentrations over 133 μmol/l. Other than the Glasgow coma score and urinary output, pre-ICU physiological data were not used in the calculation of APACHE II scores. Arterial blood gas measurements were judged to be inappropriate in some patients, and in these patients the serum bicarbonate concentration was used to calculate the physiological score [3]. One data custodian was responsible for ensuring data quality throughout the study period. The data were reviewed for internal consistency before annual lockdown, and there were no patients with missing physiological data or who were lost to mortality follow-up. The study utilised de-identified data only and was deemed to be a 'Clinical Audit' by the Hospital Ethics Committee and as such the need for formal ethics committee approval was waived.

The performance of the admission APACHE II model in predicting hospital mortality was compared with the performance of the worst 24-hour APACHE II model with respect to their discrimination ability and calibration. Because the original APACHE II prediction model did not include cardiac surgical patients, we have included only the data from noncardiac surgery ICU admissions. All patients in the database in the study period were considered, including those patients who died within 24 hours of ICU admission.

The discrimination ability of each of the scoring systems was assessed by the area under the receiver operating characteristic curve: above 90% was regarded as excellent, above 80% was regarded as good, and below 80% was regarded as poor in this study. Calibration was assessed by comparing absolute observed mortality with predicted mortality in fixed risk strata (for example 0–0.099, 0.1–0.199, and so on) using the Hosmer-Lemeshow chi-square *H *statistic. *P *< 0.05 in the Hosmer-Lemeshow chi-square *H *statistical test infers a significant departure from the null hypothesis of good calibration. The relationship between the admission APACHE II predicted hospital mortality risk and the worst 24-hour APACHE II predicted hospital mortality risk was assessed by the two-tailed Pearson correlation coefficient. The ratio of total observed to predicted mortality is the standardised mortality ratio (SMR).

The discrimination ability was further analysed for different diagnostic and patient subgroups to test the uniformity of fit of both models. The diagnostic subgroups analysed included patients with different major diagnoses such as sepsis, pneumonia, and gastrointestinal perforation or obstruction, intracranial haemorrhage, multiple trauma, cardiac arrest, and elective surgery. The patient subgroups analysed included aboriginal patients, patients transferred from another hospital, patients admitted to the ICU before or after early 1999, patients who stayed in the ICU longer than 24 hours, and patients who survived longer than 24 hours of hospitalisation. *P *< 0.05 was regarded as significant in all analyses and no adjustment was made for multiple comparisons in the subgroup analyses. All statistical analyses were performed by SPSS statistical software (version 11.0 for Windows; SPSS Inc., Chicago, IL, USA] and confidence intervals were generated by Confidence Interval Analysis (version 2.0.0; BMJ 2000, UK).

## Results

The time for collecting and checking the admission physiological data manually required an average of 5 minutes per patient (range, 3–7 minutes), and the average for the worst 24-hour physiological data was 20 minutes per patient (range, 10–40 minutes). The time required to work out the worst 24-hour APACHE II score was longer when more blood tests had been performed for the patient.

**Figure 1:**
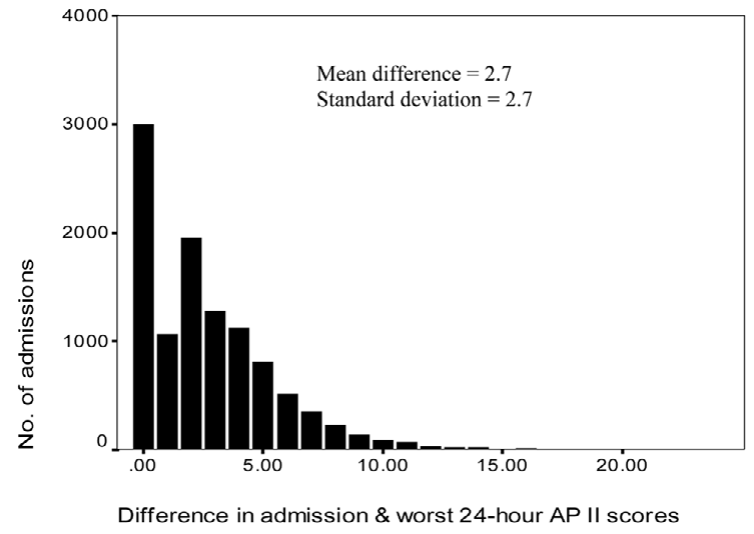
The difference in APACHE II scores using the admission and worst 24-hour physiological data. AP, Acute Physiology and Chronic Health Evaluation.

There were 11,107 noncardiac surgery ICU admissions in the 11-year period from 1 January 1993 to 31 December 2003. The characteristics of the ICU cohort are presented in Table 1. The difference in the admission APACHE II score and the worst 24-hour APACHE II score was small in most patients (Figure 1). The mean admission APACHE II score and the worst 24-hour APACHE II scores were 12.7 and 15.4, and the derived predicted hospital mortality estimates were 15.5% and 19.3%, respectively. The admission APACHE II predicted mortality and the worst 24-hour APACHE II predicted mortality were closely correlated (Pearson correlation coefficient = 0.955, *P *= 0.0001). The actual hospital mortality was 16.3%. The overall standardised mortality ratio was 1.05 (95% confidence interval [CI] = 1.00–1.10) and was 0.84 (95% CI = 0.80–0.88) using the admission APACHE II predicted mortality and the worst 24-hour APACHE II predicted mortality as the denominator, respectively.

**Figure 2:**
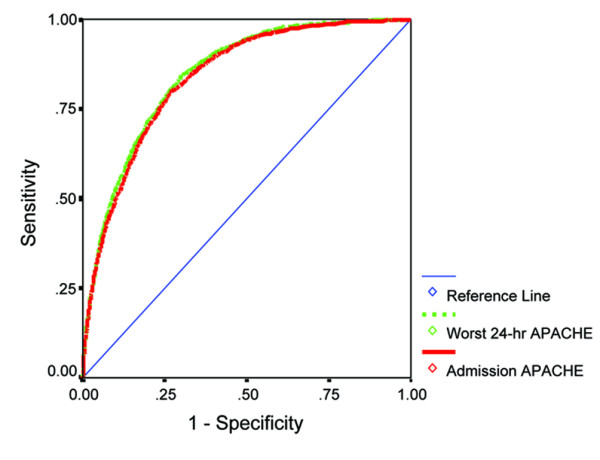
The receiver operating characteristic (ROC) curves for the admission Acute Physiology and Chronic Health Evaluation (APACHE) II model and the worst 24-hour APACHE II model in predicting hospital mortality. Area under ROC curves: worst 24-hour APACHE II model, 84.6% (95% CI = 83.7–85.5); admission APACHE II model, 83.8% (95% CI = 82.9–84.7). No significant difference between the two areas under the ROC curves (*P *= 1.00).

The overall discrimination abilities, as measured by the area under the receiver operating characteristic curve, of the admission APACHE II model (83.8%, 95% CI = 82.9–84.7) and the worst 24-hour APACHE II model (84.6%, 95% CI = 83.7–85.5) with the entire cohort were not significantly different (*P *= 1.00) (Figure 2). The discrimination abilities of the admission APACHE II model and the worst 24-hour APACHE II model were also not significantly different within all subgroups analysed (Table 2).

**Figure 3:**
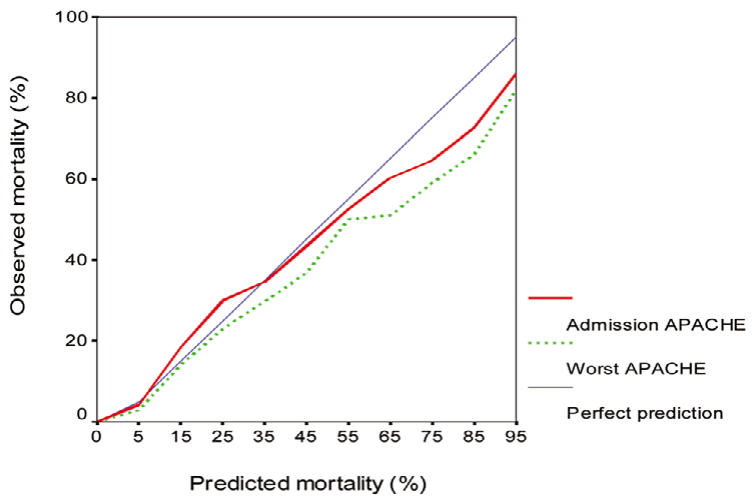
Calibration curves for the admission Acute Physiology and Chronic Health Evaluation (APACHE) II score and the worst 24-hour APACHE II score in predicting hospital mortality across different risk strata. The Hosmer-Lemeshow goodness of fit chi-square *H *statistic for the admission APACHE II predicted mortality and for the worst 24-hour APACHE II predicted mortality were 66.9 and 189.3, respectively (both *P *< 0.0001).

The Hosmer and Lemeshow goodness of fit chi-square *H *statistic was 66.7 for the admission APACHE II model and was 189.3 for the worst 24-hour APACHE II model indicating a better fit for the admission APACHE II model but both *P* values were very small (*P *< 0.0001). The calibration curve of the two APACHE II models is displayed in Figure 3 and shows the better fit of the admission APACHE II model especially in the high risk strata. The overall correct classification rate (based on classifying a patient to die if his/her predicted mortality risk exceeded 50%) for the admission APACHE II model and the worst 24-hour APACHE II model were both 85.4% (Table 3).

## Discussion

### The advantages of the admission APACHE II model

Our results showed that the performance of the admission APACHE II model is no worse than the traditional worst 24-hour APACHE II model when there are no significant missing data. These results were consistent with the results of other studies that assessed or utilised the admission APACHE II score to calculate the APACHE II predicted mortality [15-17].

The use of the admission APACHE II score to calculate the APACHE II predicted mortality (admission APACHE II model) has a few potential advantages and may represent a viable alternative to the traditional APACHE II model. First, it can assess the risk of hospital death at ICU admission, as in the MPM II_0 _and SAPS III scoring systems that assess the risk of hospital death at ICU admission [17,18]. The admission APACHE II model also shares these systems' advantages of ease of use, and, since they are independent of ICU treatment, may be more applicable for risk stratification in clinical research and triage decisions [19]. The ability of a scoring system to stratify patient risk on admission to the ICU may facilitate stratification of patients into trials that assess early interventions in critically ill patients.

Second, the data collection for the admission APACHE II model is less laborious than the worst 24-hour APACHE II model, as demonstrated in our data. It may also reduce errors because it does not require perusal of a series of values to obtain the worst score. Nevertheless, this potential advantage is important only when a computerised information system is not available and the data are collected manually.

Third, the admission APACHE II model may be a better reflection of quality of care in the ICU because risk assessment occurs before any ICU therapy is instituted [12-14].

Finally, poor calibration with the worst 24-hour APACHE II model has been reported in many studies [20-22]. Our results confirmed this problem of the worst 24-hour APACHE II model, with the predicted mortality being much higher than the actual mortality in the high-risk strata. The admission APACHE II model appeared to have reduced the overestimation of mortality in the high-risk strata and improved the calibration of the APACHE II model in the present study. However, data on calibration of the admission APACHE II model from other studies are lacking [15-17] and further studies in other settings will be needed to confirm this finding.

### Limitations of the admission APACHE II model

The admission APACHE II model is a minor modification of the worst 24-hour APACHE II model and retains many intrinsic weaknesses and problems of the worst 24-hour APACHE II model. These weaknesses include errors arising from imprecise principal diagnosis, lead time bias, and poor uniformity of fit of the model. The admission APACHE II model, as with other ICU scoring systems such as the APACHE III model, needs an accurate diagnosis to accurately predict the hospital mortality. The admission APACHE II model does not eliminate this requirement.

The performance of the worst 24-hour APACHE II model is affected by the source and timing of patient referral to the ICU, and it tends to underestimate the mortality of the patients referred from other ICUs or hospitals [23,24]. Our results were different from these reports. This may be because many patients were transferred from remote Western Australia and were not fully resuscitated when they were admitted to the ICU. The standardised mortality ratio of the patients transferred from other hospitals, based on the admission APACHE II model in this study, was closer to unity than that of the worst 24-hour APACHE II model (Table 2). The admission APACHE II model was associated with a lower lead time bias in this study. The uniformity of fit in the discrimination ability of the admission APACHE II model and the worst 24-hour APACHE II model was similarly poor in patients with sepsis, pneumonia, gastrointestinal perforation, and cardiac arrest, and also in the aboriginal patients. Both the worst 24-hour APACHE II model and the APACHE III model were not well calibrated in predicting mortality in trauma patients [23,25,26]. Our results confirmed this problem of the worst 24-hour APACHE II model, and the admission APACHE II model did not improve the performance of the worst 24-hour APACHE II model in this subgroup of patients.

### Limitations of the study

This was a single-centre study and these results may not be generalisable to other ICUs [23]. Our observation that the standardised mortality ratio calculated with the admission physiological variables was closer to unity than that calculated with the worst 24-hour values may be different in other units. Further evaluation of the admission APACHE II model in other ICUs is essential.

Also, this study did not directly compare the admission APACHE II model with other scoring systems that assess the risk of hospital mortality at ICU admission such as the MPM II_0 _and SAPS III models [17,18]. Whether the performance of the admission APACHE II model is comparable with these scoring systems remains uncertain and will be further investigated.

Critical illness is a dynamic process and therefore outcome prediction based on a single time point such as ICU admission, as in the admission APACHE II model, does not consider changes in patients' clinical status over time and their response to treatment. Serial predictions over a period of time, as in the APACHE III model, may improve prediction accuracy and clinical utilities, although acquiring these data continuously will be difficult in practice [27,28].

Finally, the admission APACHE II model, as with most other outcome prediction models, does not consider functional outcomes beyond survival [9].

## Conclusion

In conclusion, substituting the worst 24-hour physiological variables with the admission physiological variables to calculate the admission APACHE II score and the APACHE II predicted mortality does not result in significantly worse calibration or discrimination compared with the traditional APACHE II model. The admission APACHE II model represents a potential alternative model to the worst 24-hour APACHE II model in critically ill nontrauma patients.

## Key messages

• 	Modifying the APACHE II model using admission physiological variables instead of worst 24-hour physiological variables to calculate the APACHE II score and predicted mortality (admission APACHE II model) does not result in significantly worse calibration and discrimination compared with the traditional APACHE II model in critically ill nontrauma patients.

## Abbreviations

APACHE = Acute Physiology and Chronic Health Evaluation; CI = confidence interval; ICU = intensive care unit; MPM = Mortality Probability Models; SAPS = Simplified Acute Physiology Score.

## Competing interests

The authors declare that they have no competing interests.

## Authors' contributions

KMH performed the statistical analysis and drafted the manuscript. GJD initiated the original idea of the study and helped to draft the manuscript. MK, JF, and SARW helped analyse the data and draft the manuscript. KYL was the data-collection quality controller and helped to draft the manuscript. All authors read and approved the final manuscript.

## Figures and Tables

**Table 1:** Characteristics of the cohort

Variables	Mean (SD)
Age (years)	53.5 (19.5)
Male/female (%)	6,871/4,236 (61.9/38.1)
Admission source (%)	
Operating room	4,885 (44.0)
Recovery room	638 (5.7)
Emergency department	2,976 (26.8)
Ward	1,481 (13.3)
Another hospital	1,127 (10.1)
Primary organ failure (%)	
Cardiovascular	3,693 (33.2)
Neurological	3,893 (35.0)
Respiratory	2,682 (24.1)
Gastrointestinal	401 (3.6)
Renal	167 (1.5)
Metabolic	217 (2.0)
Haematological	49 (0.4)
ICU stay (days)	5.1 (7.8)
Hospital stay (days)	21.1 (29.3)
Admission APACHE II score	12.7 (7.3)
Worst 24-hour APACHE II score	15.4 (7.9)
Admission APACHE predicted mortality (%)	15.5 (19.1)
Worst 24-hour APACHE predicted mortality (%)	19.3 (22.1)
Actual ICU mortality (%)	12.0
Actual hospital mortality (%)	16.3

All data in parentheses are standard deviations unless stated otherwise. APACHE, Acute Physiology and Chronic Health Evaluation; ICU, intensive care unit; SD, standard deviation.

**Table 2:** The discriminating ability of the admission Acute Physiology and Chronic Health Evaluation (APACHE) II model and the worst 24-hour APACHE II model to predict inhospital mortality in different diagnostic and patient subgroups

Different diagnostic and patient subgroups	Number of patients	Mean area under the ROC curve (%) (95% confidence interval)^a^		Standardised mortality ratio (95% confidence interval)	
		
		Admission model	Worst 24-hour model	Admission model	Worst 24-hour model
Sepsis, pneumonia, gastrointestinal perforation or obstruction	1,474	68.3 (65.4–71.3)	68.5 (65.6–71.4)	0.94 (0.90–0.98)	0.77 (0.75–0.80)
Intracranial, subdural or subarachnoid haemorrhage	851	79.5 (76.3–82.7)	80.4 (77.2–83.5)	1.29 (1.22–1.36)	1.03 (0.98–1.08)
Multiple trauma	1,299	87.0 (84.1–89.9)	87.3 (84.4–90.1)	1.73 (1.63–1.84)	1.24 (1.17–1.31)
Cardiac arrest (nonoperative or intraoperative)	395	73.9 (69.1–78.8)	73.9 (69.0–78.8)	0.92 (0.88–0.96)	0.82 (0.79–0.85)
Elective surgery (excluding cardiac surgery)	3,012	78.6 (74.8–82.4)	80.8 (77.3–84.4)	1.04 (1.00–1.09)	0.79 (0.76–0.83)
Aboriginal patients	863	77.8 (74.2–81.4)	78.8 (75.2–82.3)	1.02 (0.95–1.09)	0.82 (0.77–0.87)
Patients transferred from another hospital	1,127	79.4 (76.3–82.4)	80.4 (77.4–83.5)	0.87 (0.82–0.92)	0.71 (0.67–0.75)
Patients admitted between 1993 and early 1999	5,553	85.4 (84.0–86.7)	86.1 (84.8–87.4)	1.05 (1.01–1.09)	0.85 (0.82–0.88)
Patients admitted between early 1999 and 2003	5,554	83.3 (82.0–84.5)	84.1 (82.8–85.3)	1.09 (1.06–1.13)	0.88 (0.86–0.91)
Patients stayed in the ICU longer than 24 hours	8,461	80.4 (79.2–81.5)	81.2 (80.1–82.3)	0.99 (0.97–1.02)	0.79 (0.77–0.81)
Patients survived longer than 24 hours of hospitalisation	10,733	82.2 (81.1–83.2)	83.0 (82.0–84.0)	0.93 (0.91–0.95)	0.74 (0.73–0.76)

^a^There was no significant difference in the areas under the receiver operating characteristic (ROC) curves between the admission APACHE II model and the worst 24-hour APACHE II model (*P *= 1.00).

**Table 3:** Classification table for the admission Acute Physiology and Chronic Health Evaluation (APACHE) II model and the worst 24-hour APACHE II model to predict hospital mortality

Observed hospital mortality	Predicted hospital mortality		
	
	No (*n*)	Yes (*n*)	% correct
Using the worst 24-hour APACHE II model			

No	8,899	394	95.8
Yes	1,229	585	32.2
Overall percentage			85.4

Using the admission APACHE II model			

No	8,966	327	96.5
Yes	1,293	521	28.7
Overall percentage			85.4

The cutoff value is 0.50.
